# Observations of Turbulence in Free Atmosphere by Balloon-Borne Sensors

**DOI:** 10.3390/s18103273

**Published:** 2018-09-28

**Authors:** Lesong Zhou, Zheng Sheng, Qixiang Liao

**Affiliations:** 1 College of Meteorology and Oceanography, National University of Defense Technology, Nanjing 211101, China; 17327729696@163.com (L.Z.); liaoqixiang2013@126.com (Q.L.); 2 Collaborative Innovation Center on Forecast and Evaluation of Meteorological Disasters, Nanjing University of Information Science and Technology, Nanjing 210044, China

**Keywords:** turbulence, balloon-borne sensors, Thorpe analysis

## Abstract

In recent years, Thorpe analysis has been used to retrieve the characteristics of turbulence in free atmosphere from balloon-borne sensor data. However, previous studies have mainly focused on the mid-high latitude region, and this method is still rarely applied at heights above 30 km, especially above 35 km. Therefore, seven sets of upper air (>35 km) sounding data from the Changsha Sounding Station (28°12′ N, 113°05′ E), China are analyzed with Thorpe analysis in this article. It is noted that, in the troposphere, Thorpe analysis can better retrieve the turbulence distribution and the corresponding turbulence parameters. Also, because of the thicker troposphere at low latitudes, the values of the Thorpe scale $L_{T}$ and turbulent energy dissipation rate ε remain greater in a larger height range. In the stratosphere below the height of 35 km, the obtained ε is higher, and Thorpe analysis can only be used to analyze the characteristics of large-scale turbulence. In the stratosphere at a height of 35–40 km, because of the interference of sensor noise, Thorpe analysis can only help to retrieve the rough distribution position of large-scale turbulence, while it can hardly help with the calculation of the turbulence parameters.

## 1. Introduction

Atmospheric turbulence is an important research hotspot in the field of atmospheric science. It is of great importance to understand the energy budget, momentum transfer, and trace gas distribution in the atmosphere [1]. Turbulence parameters such as eddy diffusivity and the dissipation rate of turbulent kinetic energy are key elements used for modeling the vertical distribution of trace constituents in the atmosphere, as well as for assessing the exchange between the troposphere and stratosphere [2]. The full understanding of turbulent mixing is beneficial to both climate prediction and weather forecasts [3]. Also, the research on turbulence in the troposphere and stratosphere helps to increase the knowledge of the middle atmosphere and improve the ability to predict atmospheric turbulence. This is significant for the safety of aircraft in near-space. After decades of efforts, great progress has been made in the study of mixing in the atmospheric boundary layer [4], but the study of free atmospheric turbulence is lagging behind, relatively speaking [5]. Our lack of understanding of the latter is the result of the fact that the detection of free atmosphere is limited by its quality (height and resolution of detection) and quantity [6,7], and that the free atmospheric turbulence is episodic [8,9]. In order to solve the problems above, it is necessary to explore an accessible method to detect turbulence characteristics despite the difficulties [10]. Radar detection is easily interfered with by various factors [11], while rocket detection and aircraft detection is expensive [12,13]. Balloon sounding seems to be a more effective method by comparison. Balloon sounding is not only able to perform detection in situ with balloon-borne sensors, but also has a better resolution [14]. In order to retrieve the characteristics of turbulence of free atmosphere from balloon sounding data, many researchers have used Thorpe analysis, which was originally applied to the study of ocean mixing, to analyze balloon sounding data [15,16,17,18,19,20,21,22]. This innovative method provides a new approach to the study of free atmospheric turbulence.

Thorpe analysis is a method to obtain the turbulence parameters by calculating the difference between the detected potential temperature profile or potential density profile and the reference profile obtained by sorting the data [15,16]. In the free atmosphere, with the use of balloon-borne sensors to detect the temperature, pressure, and humidity, we can get the potential temperature (θ) profile. However, the profile is unstable and contains a large number of inversions, namely some areas with a large θ in the lower layer and small θ in the higher layer. In order to obtain a stable profile, the potential temperature will be arranged in ascending order. We arrange the potential temperature from small to large according to the height from low to high. In this process, supposing that θ at $z_{n}$ needs to be moved to $z_{m}$ after rearrangement, then the Thorpe displacement $D=z_{n}-z_{m}$. The root mean square of all D in the entire inversion is the Thorpe scale $L_{T}$, which characterizes the scale of the inversion. $L_{T}$ is related to the Ozmidov scale $L_{O}$, which is an important scale to describe turbulence characteristics; that is,

(1) $$L_{O}=cL_{T}$$

where c is the empirical constant, $L_{O}={\left(\frac{\varepsilon }{N^{3}}\right)}^{\frac{1}{2}}$, ε is the turbulent energy dissipation rate, and N is the buoyancy frequency. Although there are some uncertainties in c [16,17,18], the proportionality between $L_{T}$ and $L_{O}$ is well established and beyond dispute [19]. Then, we can obtain

(2) $$\varepsilon =C_{K}L_{T}^{2}N^{3}$$

where $C_{K}=c^{2}$. According to previous studies [20], in this article, we take the value of $C_{K}$ as 0.3. In order to verify the validity of Thorpe analysis, Gavrilov et al. compared Thorpe scales obtained by Thorpe analysis and measured by simultaneous aircraft and found a good agreement between the two [8]. In Clayson and Kantha’s research, it is discovered that the dissipation rate of turbulence obtained by Thorpe analysis is basically consistent with that measured by radar [9]. All this proves that Thorpe analysis is an effective method to obtain turbulent parameters. In recent years, some related studies have been published. In these studies, Thorpe analysis has been applied to sounding data in different regions of the earth, which greatly enriched the application results of Thorpe analysis. In this process, this method has also been constantly improved and developed [21,22,23]. Wilson et al. introduced the method of removing noise through the sample range, which has a good effect and is also a commonly-used denoising method [21]. The basic idea is that, if we suppose that an inversion is caused by turbulence, the variation range of θ in the interior must be greater than that of θ caused by pure noise under the same condition. Therefore, an inversion whose range is less than the range caused by pure noise can be removed as a result of noise. Generally, most of the inversion will be removed, while a small part of the inversion, which indeed includes turbulence, will be retained.

In previous studies, most of the data analyzed by Thorpe analysis are detected from the mid-high latitudes regions [8,9,24,25], and of course tropical regions [20,26], but the data from the low latitude regions outside the tropics seem to not have been analyzed yet. In addition, the maximum height of the previous study was only about 30 km because of the limitation of the balloon rise height [9,25,26]. Over 30 km, the effect of Thorpe analysis remains unknown. Therefore, in this article, Thorpe analysis is used to analyze the seven sets of upper air (>35 km) sounding data from Changsha Sounding Station (28°12′ N, 113°05′ E) in China. The top height that the sounding balloon reaches in each group is greater than 35 km, and the results are discussed. Through this research, we hope to improve our knowledge of the characteristics of the free atmospheric turbulence in the low-altitude regions aside from the equatorial area and of the application effect of Thorpe analysis in the height range of 30–40 km.

In Section 2, a detailed description of the data used and data processing methods is provided. The statistics and analysis of the results are presented in Section 3. In Section 4, we discuss the influence of noise, and Section 5 is the main conclusion.

## 2. Dataset and Data Processing

### 2.1. The Datasets

**Figure 1:**
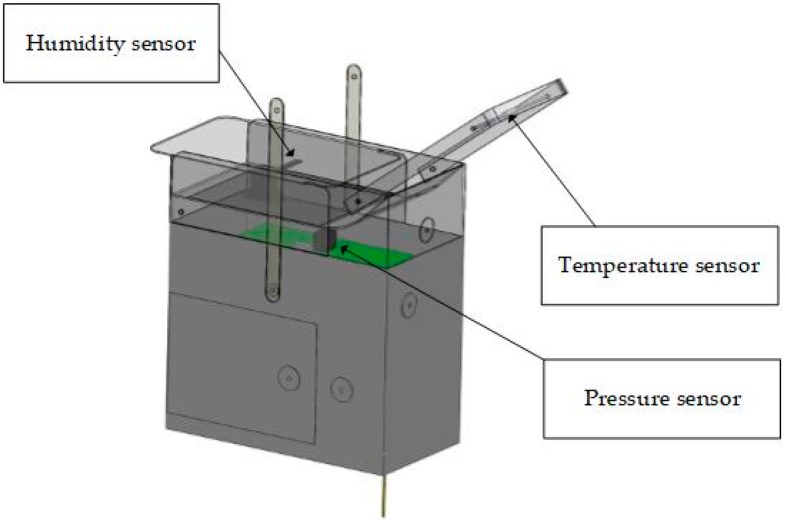
The measurement set up.

The data used in this article are seven sets of sounding balloon data from Changsha Sounding Station in China from January 2013 to January 2016. The sensors used in this article are the rod-shaped thermistor (temperature), silicon piezoresistive pressure sensor (pressure), and thin-film moisture-sensitive resistance (relative humidity). The detailed measurement set-up and specifications of accuracy are shown in Figure 1 and Table 1, respectively. The detection frequency is 1 Hz. Because the average rise speed of the seven balloon is about 9 m/s, the average vertical resolution of the sounding balloon is about 9 m/s (7.69–10.67 m/s). Besides the top height of the sounding balloons released in January 2013, which only reached 36 km, the top height of the other sounding balloons is greater than 40 km. The stratopause height is about 50 km [27]; this allows us to obtain vertical meteorological parameters throughout the troposphere as well as most of the stratosphere. The original parameters obtained by sounding balloons are temperature, pressure, and relative humidity. The height, potential temperature, and other parameters are retrieved on the basis of temperature, pressure, and relative humidity.

In order to obtain the atmospheric background state during sounding, we use ERA-Interim (ECMWF interim Re-Analysis) data to calculate the background atmospheric environment parameters. ERA-Interim is a global atmospheric reanalysis from 1979 to present, produced by a numerical weather prediction model run at the European Centre for Medium-Range Weather Forecasts [28]. The highest horizontal resolution of ERA-Interim is 0.25° × 0.25°, with a temporal resolution of 6 h. In the vertical direction, it is divided into 37 layers. In addition to providing data of temperature, pressure, and humidity, ERA-Interim can also provide wind field data. After obtaining the data of the grid point that has the smallest time and spatial interval with the balloon release site, we can calculate two important parameters for measuring atmospheric stability: windshear and gradient Richardson number (Ri) [29,30],

(3) $$Ri=\frac{\frac{g}{\bar{\theta }}•\frac{\partial \bar{\theta }}{\partial z}}{\left[{\left(\frac{\partial \bar{u}}{\partial z}\right)}^{2}+{\left(\frac{\partial \bar{v}}{\partial z}\right)}^{2}\right]}$$

### 2.2. Calculation of Height and Potential Temperature

The height *z* is inversed mainly by the barometric height formula:

(4) $$\Delta z_{i}=\frac{RT_{i}}{g}\ln\frac{P_{i}}{P_{i+1}}$$

where R is the specific gas constant, g is the acceleration of gravity, T is the detection value of temperature, P is the detection value of atmospheric pressure, and Δz is the height difference between two adjacent detection points. Generally, in the ascent of the balloon, the air pressure becomes lower and lower. However, at some locations, the upper layer pressure value detected by the balloon is greater than that of the lower layer. This leads to the result that the height of the upper layer obtained by inversion is less than the height of the lower layer, which is obviously unreasonable. Therefore, in the process of retrieving *z*, based on the method of Wilson et al. [31], we processed the pressure data with a least square cubic spline approximation to obtain the approximate *P^A^*. By substituting $P^{A}$ into (4), the height obtained is monotonically increasing, which can better reflect the actual change of height of the balloon.

As with z, the potential temperature θ is also calculated based on the temperature detection value T and pressure detection value P. The calculation formula is

(5) $$\theta =T{\left(\frac{P_{0}}{P}\right)}^{\frac{R}{c_{pd}}}$$

where $P_{0}$ is the pressure at the reference level and $c_{pd}$ is the specific heat for dry air.

As the influence of the water vapor is quite small in the unsaturated air [23], and all the sounding balloons used in the study did not pass through the saturated air, the effect of saturated water vapor was not taken into consideration for the calculation of the θ.

### 2.3. Calculation of Turbulent Parameters

Thorpe analysis is a method to infer atmospheric turbulence parameters based on the inversion of θ. The specific steps include the calculation of the Thorpe scale $L_{T}$, removal of noise influence, and the transformation of the Thorpe scale with other turbulence parameters.

#### 2.3.1. Calculation of $L_{T}$

The calculation method of $L_{T}$ is mentioned in the introduction. By rearranging the potential temperature profile calculated from T and P, we can obtain a new θ profile that monotonically increases with height. Assuming that θ at $z_{n}$ is moved to $z_{m}$ after rearranging, the height difference between them $D=z_{n}-z_{m}$ is called Thorpe displacement. For an independent inversion region, the displacement at the bottom of the reversion is negative while that at the top is positive, and the sum of all D in its interior is zero. Supposing $z_{a}$ and $z_{b}$ are the upper and lower boundaries of inversion, D meets the following condition,

(6) $$\left\{ \begin{array}{l} \sum_{i=a}^{i=k}D_{i}=0,(k=b)\\ \sum_{i=a}^{i=k}D_{i}<0,(a\leq k\leq b) \end{array}\right.$$

Thus, it is also through this feature that we determine the boundaries of the inversion regions. After the boundary is determined, the Thorpe scale $L_{T}$ is obtained by calculating the root-mean-square (rms) of all Thorpe displacements in the inversion.

#### 2.3.2. Removal of Noise Influence

The Thorpe analysis is a method of inferring the characteristics of atmospheric turbulence based on the inversions of θ (referred to as inversions). However, the inversions detected by the sensors are caused not only by the natural environment factors such as turbulence, but also by the noise generated by the instrument. Regardless of the existence of turbulence, instrument noise (referred to as noise) always exists. Assuming that there is no change in the θ of the environment in the process of detection, noise can still cause random fluctuations in the detected θ, resulting in false inversion. However, this is not the inversion caused by atmospheric motion, and so the noise has a huge impact on Thorpe analysis. To avoid noise interference, the inversion purely caused by noise must be screened out and removed to get the inversion caused by atmospheric motion. In this article, we mainly use the method of Wilson et al. [21] to remove the influence of noise by the variation range of the θ in the inversion. Specific steps are as follows:

Firstly, we estimate the standard deviation of instrument noise. According to the study of Gavrilov et al. [8], the structure function method can be used to estimate the instrument noise variance. Assuming that the measured value of physical quantity v(t) is composed of two parts, the true value of physical quantity v(t) and the random error n(t),

(7) v(t)=V(t)+n(t)

**Figure 2:**
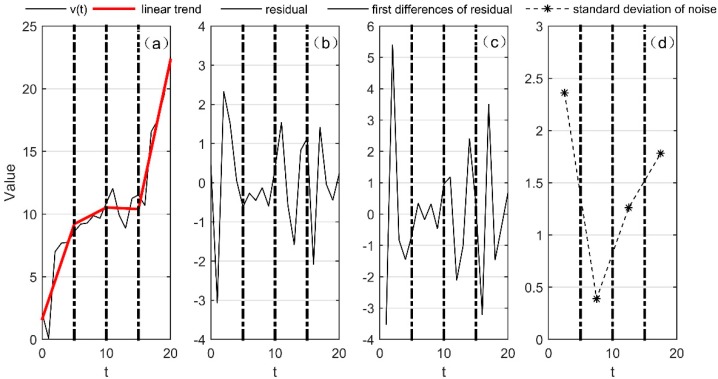
The specific steps of estimating the noise standard deviation.

Assuming the error is completely random, then the structure function is

(8) $$D(t^{\prime })=\bar{{\left[v(t+t^{\prime })-v(t)\right]}^{2}}=2\left[\bar{V^{2}}-\bar{V(t+t^{\prime })V(t)}+\bar{n^{2}}\right]$$

where $t^{\prime }$ is the time interval between two measurements. When the detected physical quantity has no obvious trend and $t^{\prime }\to 0$,

(9) $$D(t^{\prime })=2\bar{n^{2}}=2\sigma_{n}^{2}$$

where $\sigma_{n}$ is the noise standard deviation [32]. However, the detected temperature and pressure change as the height changes. Thus, in order to meet the condition of (8), the trend of the data needs to be removed. The specific steps of estimating the noise standard deviation of the instrument are shown in Figure 2. Supposing the black line in (a) is the original data *v*(*t*), we create an interval of 5 points in *v*(*t*), then gives a linear fit for each interval and the data trend as shown by the red line in (a). We use the original data minus the trend and get the residual error as shown in (b). We obtain the first difference through calculation, as shown in (c). Finally, we calculate the standard deviation of the residual error’s first difference for each interval and divide it by $\sqrt{2}$ and get the noise standard deviation of the original data for each interval, as shown in (d).

In this article, *T* and *P* are the original detection data, and θ is calculated by (5), so the standard deviation of the potential temperature noise $\sigma_{\theta }$ needs to be calculated by

(10) $$\sigma_{\theta }=\theta \sqrt{{\left(\frac{\sigma_{T}}{T}\right)}^{2}+{\left(\frac{2}{7}\frac{\sigma_{P}}{P}\right)}^{2}}=\sqrt{{\left(\frac{P_{0}}{P}\right)}^{\frac{4}{7}}\left[\sigma_{T}^{2}+T^{2}{\left(\frac{2}{7}\frac{\sigma_{P}}{P}\right)}^{2}\right]}$$

This is done after calculating the standard deviation of the temperature noise $\sigma_{T}$ and the standard deviation of the pressure noise $\sigma_{P}$. With the gradual increase of height, the pressure will gradually decrease. Especially when the height is over 30 km, the pressure drops sharply and the value of $\frac{P_{0}}{P}$ rises sharply, which results in the rapid increase of $\sigma_{\theta }$ and also means the instrument noise reaches a higher level. This phenomenon has a great influence on Thorpe analysis, and we will explain this in detail in Section 4.

Secondly, we calculate the trend-to-noise ratio (TNR); TNR is a key parameter for quantifying the impact of measurement noise in a sorting procedure. It is defined as

(11) $$\bar{\zeta }=\frac{\theta_{n}-\theta_{1}}{\sigma_{\theta }(n-1)}$$

when TNR is less than 1, we think that Thorpe analysis is seriously disturbed by noise and needs to be processed with filtering and undersampling. In this article, all the original TNRs of the seven sets of data are more than 1 (1.4–1.9); thus, there is no need to do the above process.

**Figure 3:**
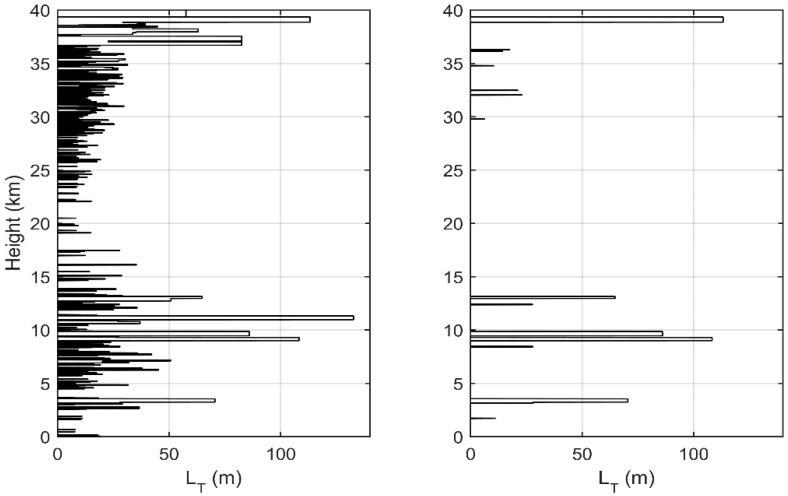
Thorpe length before denoising (**left**) and after denoising (**right**).

Thirdly, we remove the inversion purely caused by instrument noise. The basic principle is that if the θ inversion, whose size is n (n represents the number of probing points in inversion), is caused by turbulence, its internal θ range $W_{\theta }(n)$ after the trend is removed will be significantly larger than the range $W_{N}(n)$ that has the same size caused purely by noise (*N*, namely noise). When n is determined, the size of $W_{N}(n)$ is uncertain (see Wilson et al. [21], Figure 3). For convenience, $W_{N}^{99}$ or $W_{N}^{95}$ (99 and 95 indicate that, for a particular random sample, the probability of its range $W_{N}(n)$ less than $W_{N}^{99}$ or $W_{N}^{95}$ is 99% or 95%) is generally used as the threshold of $W_{\theta }(n)$. In this article, $W_{N}^{99}$ is taken as a threshold. Wilson et al. [21] gives the value of $W_{N}^{99}(2\leq n\leq 1000)$ of the standard normal distribution through Monte Carlo simulation. When the standard deviation of the potential temperature noise is $\sigma_{\theta }$, the following inversion will be removed as a result of noise:

(12) $$W_{\theta }(n)<W_{N}^{99}(n)\sigma_{\theta },$$

As shown in Figure 3, the distribution of $L_{T}$ is based on the data obtained on 28 October 2015. Before denoising, 32.6% of the regions showed the presence of inversion. Except for a few real inversions, most of the other inversions are shorter in length, smaller in thickness, and denser, which can clearly reflect the interference of noise. After denoising, the distribution of inversions obviously become sparse, and only 5.1% of the regions are inverted. About 95.7% of the inversions are considered to be caused purely by noise and are eliminated. At about 4–8 km and about 25–30 km, the denser inversions before denoising turn into large blank areas because of the relatively stable atmospheric condition, and noises become the main reason for the inversions. That is why denoising is necessary both in the stratosphere and in the troposphere with the application of Thorpe analysis. After screening, the retained inversion mainly concentrates in the area of 8–13 km, with a relatively large thickness and Thorpe scale. The Thorpe scale is more than 100 m at 9 km (consistent with the research of [19]), and the thickness is also in the same order of magnitude. Under the tropopause, wind speed is large [5,20], which is conducive to the generation of turbulence, and so $L_{T}$ also has a corresponding large value in this region. In the middle of the stratosphere, the wind speed becomes smaller, yet the wind shear is larger, which is also beneficial to turbulence generation; this, plus the influence of noise, makes a larger $L_{T}$ appear in this area.

#### 2.3.3. Turbulence Parameters Calculated with $L_{T}$

**Figure 4:**
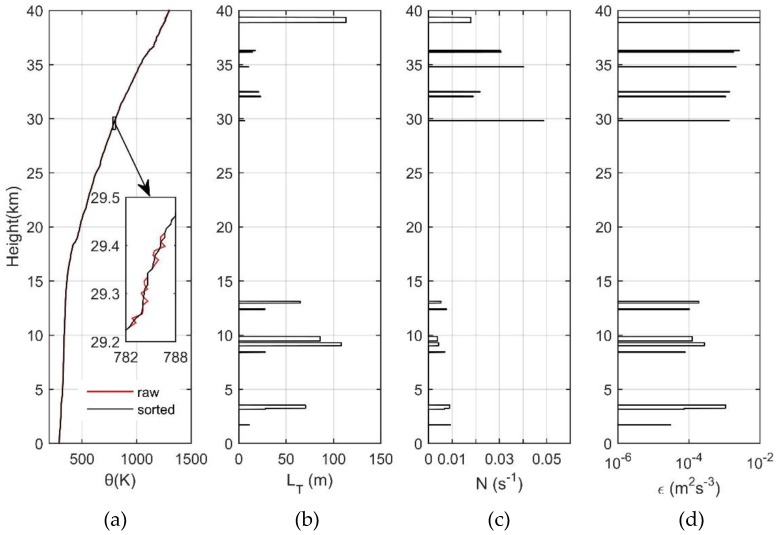
(**a**) Potential temperature; (**b**) Thorpe scale; (**c**) buoyancy frequency; and (**d**) turbulent energy dissipation rate.

Thorpe analysis was first used in studies on oceans and lakes. The Thorpe length $L_{T}$ reflects the scale of seawater overturning caused by turbulence and other factors, and the Ozmidov scale $L_{O}$ reflects the maximum scale of turbulence in stratified fluid. According to (1) and (2), we can associate the observed potential temperature overturning with the important physical quantities $L_{O}$ and ε that describe the turbulence characteristics. In the actual calculation, in order to compare the ε obtained in the troposphere and that in the stratosphere with Thorpe analysis, N takes the average value of the entire inversion region, and so the obtained ε is also the average value of the entire inversion region. In the region where there is no inversion, $L_{O}$, N, and ε are set to zero and are not compared in the troposphere and in the stratosphere. Also shown in Figure 4 is the vertical distribution of the four main physical parameters based on the data of 28 October 2015. (a) reflects the change of θ with changing height. On the whole, the θ profile before and after the sorting basically coincides and shows a gradual upward trend as height increases, rising from about 300 K on the ground to about 1300 K at 40 km. Below the tropopause (about 18 km), the potential temperature increases slowly as altitude increases. Above the tropopause, however, the potential temperature increases abruptly with height increase. In the partial enlarged view, we can clearly see the difference between the before and after arrangement. There are obvious inversions of the profile before the arrangement, which may be caused by the atmospheric turbulence layers. On the other hand, this may be caused by noises; (b) is exactly the same as Figure 3, reflecting the vertical distribution of $L_{T}$; (c) is the buoyancy frequency N. Below the tropopause, the gradient of θ is small and N is generally less than 0.01 s^−1^. Above the tropopause, the gradient of θ becomes significantly larger and N is generally larger than 0.02 s^−1^. According to Equation (2), we know that ε is proportional to the square of $L_{T}$ and proportional to the cube of N. Therefore, the value of ε mainly depends on the value of N. This leads to a smaller value of ε under the tropopause of about 10^−^^4^ (m^2^ s^−^^3^) and bigger values of ε above the tropopause, most of which are over 10^−4^ (m^2^ s^−3^), and some even reach 10^−2^ (m^2^ s^−3^) as shown in (d).

## 3. Analysis and Statistics of Results

**Figure 5:**
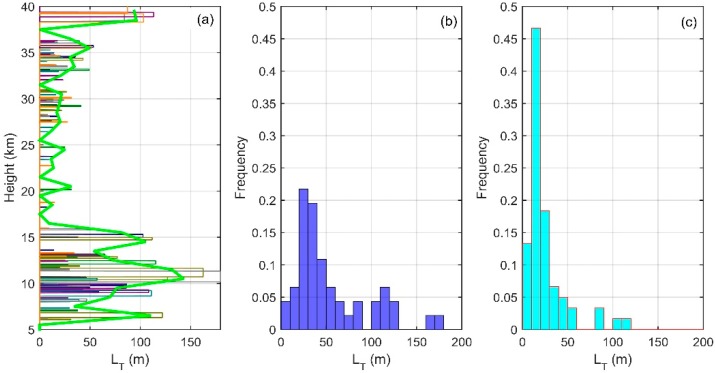
Vertical distribution of $L_{T}$ (**a**) and frequency distribution of $L_{T}$ in the troposphere (**b**) and in the stratosphere (**c**).

**Figure 6:**
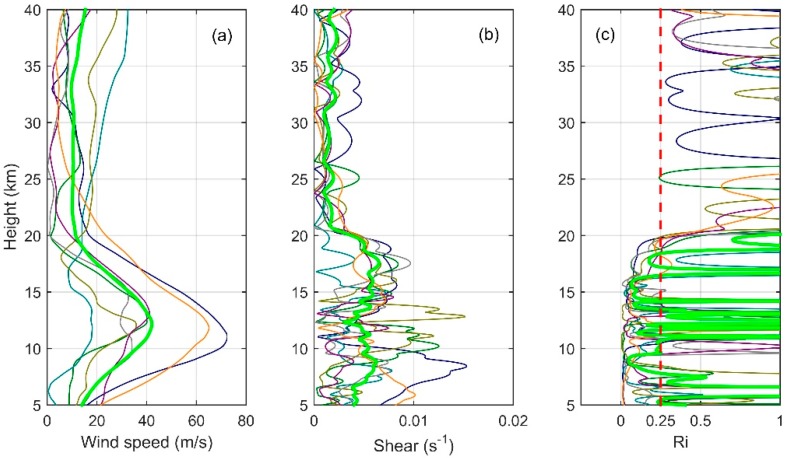
Wind profiles (**a**); windshear profiles (**b**); and Ri profiles (**c**) obtained from ERA-Interim data.

In order to get the distribution characteristics of turbulence parameters obtained with Thorpe analysis in the troposphere and stratosphere, we conducted analysis and statistics on the seven sets of sounding data. Figure 5 shows the vertical distribution of $L_{T}$ (a) and frequency distribution of $L_{T}$ in the troposphere (b) and in the stratosphere (c) obtained from sounding data. Figure 6 shows the wind profiles (a), windshear profiles (b), and Ri profiles (c) obtained from ERA-Interim data. In Figure 5a and Figure 6, seven sets of data are represented by thin lines of different colors, and the average of seven sets of data is shown by bold green lines. As the Thorpe analysis is mainly applied in the free atmosphere [16,21], we do not discuss the state of 0–5 km. It can be seen from the figure that the areas with large $L_{T}$ mainly lie in the ranges 5–16 km and 38–40 km. In the height range of 7–13 km, $L_{T}$ is featured with a larger value in the middle part (9–11 km) and a smaller value at the top and bottom. In the middle, the maximum value of $L_{T}$ is about 180 m, and this part is also the area where the inversion is more concentrated. This shows that the turbulence activity is frequent, with a large scale in this area. As can be seen from Figure 6, this is mainly because, above the boundary layer and below the tropopause, the wind shear rate is large and the Ri is small. The Ri at most regions is less than 0.25, which means that the atmosphere is in an unstable state and is favorable for the development of turbulence. In the range of 38–40 km, the maximum value of $L_{T}$ exceeds 100 m, but unlike the range of 7–16 km, this part has a smaller number of inversions. This is mainly because of the fact that, above the tropopause, the state of the atmosphere tends to be stable and the turbulence activity is not as frequent as in the troposphere, so this part is less inverted. However, Thorpe analysis is susceptible to noise interference at higher altitudes, thus there is a larger value of $L_{T}$ in this part, we will explain this in detail in Section 4. The range of 16–31 km is where the smaller $L_{T}$ mainly concentrates and where the inversions are relatively sparse, because this range has a larger gradient Richardson number, more stable stratification, and because it is not easy to turn the atmosphere from the laminar state to turbulent state. Especially in the range of 20–23 km, there is almost no inversion. The range is also where the quasi-zero wind layer (18–25 km) appears in China [33]. Wind shears and gravity waves are the main causes of turbulence, so the atmospheric environment of the quasi-zero wind layer is not conducive to the generation and development of turbulence. Even though there was a small scale of turbulence, this is beyond the sounding scope of the sensors, thus no inversion was detected by the sensors in this range.

The (b) and (c) in Figure 5 shows the frequency distribution of $L_{T}$ in the troposphere and stratosphere in different ranges, and the interval of the histogram’s abscissa is 10 m. Except for some extremely large values, the frequency distribution of the $L_{T}$ value in the troposphere is more even than that in the stratosphere, mainly concentrated in the range of 20–50 m. The maximum frequency of about 0.2 appears in the range of 20–30 m. This is mainly because of the more intense tropospheric wind shear and the larger turbulence size. The value of $L_{T}$ in the stratosphere is relatively small, mainly concentrated in the 0–30 m range. The largest frequency, over 0.45, appears in the range of 10–20 m. In the range of 20–80 m, the frequency of the $L_{T}$ in the stratosphere is less than that in the troposphere. This shows that, in the stratosphere, because of the relative stability of the atmospheric environment, the size of the turbulence is relatively small and the inversion detected by the sensors is also concentrated in the smaller size range. However, in both the stratosphere and the troposphere, there are a few large values that deviate significantly from the relatively concentrated regions. This may because of the existence of large-scale turbulence caused by inhomogeneity of turbulence [34,35]. It might also be because the sensors were limited by the resolution and could not distinguish turbulent layers that are close to each other and mistook them for an entire turbulent layer. That is why several values of $L_{T}$ are obviously larger than the average state. Also, this may be caused by noise, whose effect will be discussed in Section 4.

**Figure 7:**
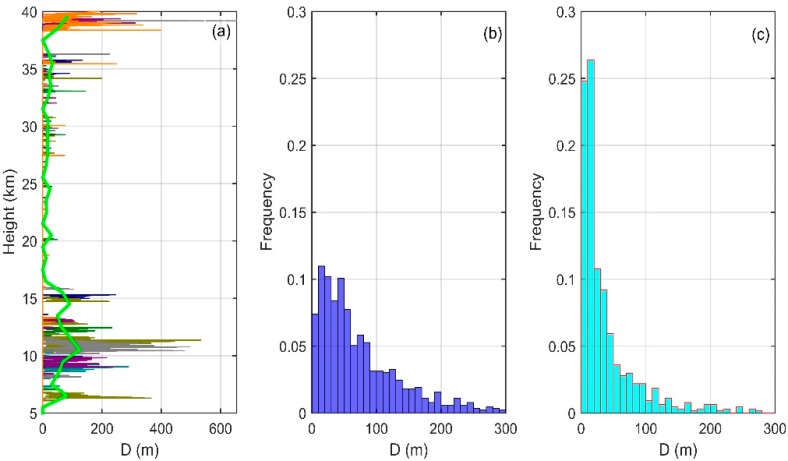
Vertical distribution of *D* (**a**) and frequency distribution of *D* in the troposphere (**b**) and in the stratosphere (**c**).

Figure 7 shows the vertical distribution of *D* (a) and frequency distribution of *D* in the troposphere (b) and in the stratosphere (c) (for convenience of observation, a few displacement lengths whose value are greater than 300 m in the frequency distribution are not shown). The vertical distribution of Thorpe displacement and vertical distribution of $L_{T}$ are basically consistent with each other, both showing a relatively large value in the troposphere and a relatively small value in the stratosphere. As $L_{T}$ is the root mean square of the Thorpe displacements in a complete inversion, the max value of the Thorpe displacement is obviously greater than that of the $L_{T}$. There are many Thorpe displacements with a length greater than 200 m in the range of 8–16 km and 38–40 km, which are the main reason for the larger $L_{T}$ in this height range. In the figure of frequency distribution, both in the troposphere and stratosphere, a larger frequency appears in the smaller range (0–50 m), with the largest frequency in the range of 10–20 m. Then, the frequency decreases with the increase of the height range. However, in the troposphere, the frequency of Thorpe displacement in the range of 0–50 m is about 0.5, while in the stratosphere, only in the range of 0–20 m, the frequency is over 0.5. From the analysis above, we can see that, in the rearrangement process of θ in the troposphere, the exchange distance of θ is far, while in the stratosphere, most of the θ exchanges occur only between adjacent 1–2 probing points, and long-distance exchanges are relatively rare.

**Figure 8:**
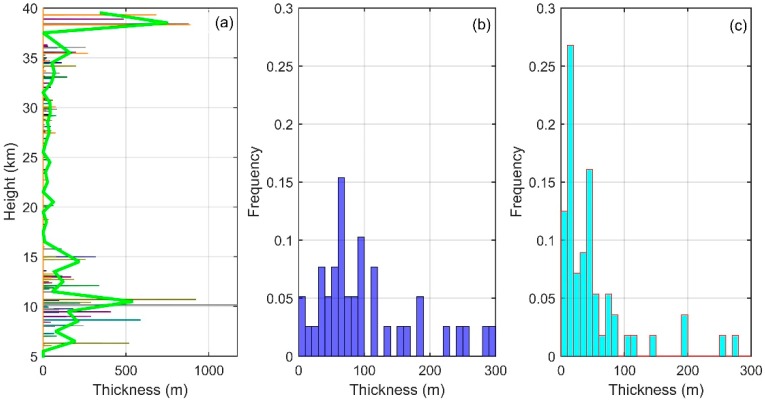
Vertical distribution of inversion thickness (**a**) and frequency distribution of inversion thickness in the troposphere (**b**) and in the stratosphere (**c**).

Figure 8 is the vertical distribution and frequency distribution of the inversion thickness (in the figure of vertical distribution, the height corresponding to the thickness is the lowest height of the inversion; as in Figure 7, several values of thicknesses more than 300 m are not shown in the frequency distribution). As can be seen from the figure, the vertical distribution of vertical thickness and the vertical distribution of displacement as well as the vertical distribution of $L_{T}$ are basically the same. When the inversion thickness is larger, $L_{T}$ is also larger. For example, the thickness of maximum inversion is nearly 400 m (10 km), and its corresponding $L_{T}$ reaches nearly 100 m. However, in the figure of frequency distribution, the difference between the troposphere and the stratosphere is obvious. In the stratosphere, as to what appears in the frequency distribution of $L_{T}$ and Thorpe displacement, the frequency of the thickness in the range of 0–20 m (about 1–2 times the average resolution), at about 0.4, is the largest. In addition, most of the thickness is distributed in the smaller range (0–50 m). In the troposphere, the frequency of the thickness in the range of 30–100 m is larger. This shows that, in the troposphere, the thickness of the turbulent layer is generally larger, so it can be better recognized by the sensors. However, in the stratosphere, the thickness of the turbulent layer is significantly smaller. One individual turbosphere whose thickness is less than the sensor resolution cannot be detected by the sensor. For several adjacent turbospheres, although their thickness is less than the sensor resolution, they cannot be distinguished by the sensor because of its resolution limit. In this situation, the sensor takes several small turbospheres as one big turbosphere, leading to a concentrated distribution in the range of 0–20 m of thickness.

**Figure 9:**
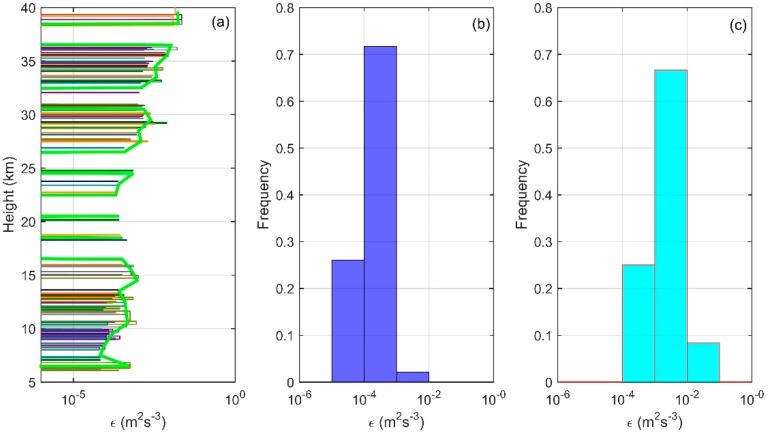
Vertical distribution of ε (**a**) and frequency distribution of ε in the troposphere (**b**) and in the stratosphere (**c**).

Figure 9 is the vertical distribution and frequency distribution of the turbulent energy dissipation rate. In the troposphere, similar to $L_{T}$, the ε also appears to be larger in the middle troposphere and smaller in the upper and lower parts. In the middle of the troposphere, the value of ε is close to 10^−3^ m^2^ s^−3^, and in the other height range, the lower value is between 10^−4^ m^2^ s^−3^ and 10^−5^ m^2^ s^−3^. In the range of 20–23 km, as $L_{T}$ is 0, the ε is 0. In the stratosphere, the ε increases step by step, increasing from a lower value 10^−4^ m^2^ s^−3^ to about 10^−2^ m^2^ s^−3^. On the histogram of the frequency distribution, the difference between the stratosphere and the troposphere is more obvious. In the troposphere, the range of larger frequency is 10^−5^–10^−3^ m^2^ s^−3^, accounting for about 97% of the total. The remaining 3% of the ε is between 10^−3^–10^−2^ m^2^ s^−3^. In the stratosphere, the range of larger frequency is 10^−4^–10^−2^ m^2^ s^−3^, which accounts for about 91% of the total. The rest of the ε is distributed between 10^−2^–10^−1^ m^2^ s^−3^. In general, both in the troposphere and the stratosphere, the ε is only distributed in three orders of magnitude, and the distribution trend is generally consistent. The histogram of the stratosphere looks like the histogram of the troposphere shifted toward a larger value by one order of magnitude, which shows that the ε in the stratosphere obtained with Thorpe analysis is generally a larger order of magnitude than that of the troposphere. From Formula (2), it can be seen that the value of the ε is not only influenced by the $L_{T}$, but also by the buoyancy frequency N. In the troposphere, the value of N is generally less than 0.01 s^−1^, and the change of the magnitude of epsilon is mainly affected by $L_{T}$, while in the stratosphere, the value of N is generally larger than 0.01 s^−1^, about 0.02 s^−1^, making the value of ε larger. This shows that, in the condition of a relatively stable environment of stratospheric atmosphere, it is not easy to generate turbulence. Even if it is generated, the scale is very small. However, once the turbulence is generated, the surrounding stable atmosphere will dissipate the energy of turbulence quickly to bring this area back to the stable state; thus, there is a larger turbulence energy dissipation rate.

## 4. Discussion

**Figure 10:**
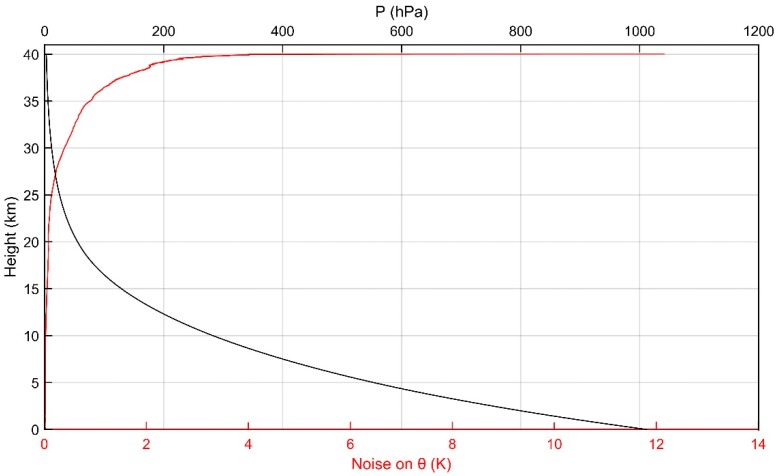
Vertical distribution of noise standard deviation and pressure.

As can be seen from Figure 6, in the area above 30 km, smaller wind shear and larger Ri indicate that the atmospheric environment in this region is stable, and it is unlikely that there will be some large turbulent layers. At the same time, we know from Formula (10) and Figure 10 that the instrument noise is very strong in this area. Although in this article we have used the method of Wilson et al. [21] to denoise the data, it cannot completely eliminate the effect of noise. According to the hypothesis of Wilson et al. [21], the inversion of potential temperature may be caused either by turbulence and instrument noise or purely by instrument noise. Generally, the range of the inversion of the potential temperature $W_{\theta }(n)$ caused by turbulence and instrument noise is much bigger than the range of the inversion of potential temperature $W_{N}(n)$ purely caused by instrument noise. Therefore, the inversion whose $W_{\theta }(n)$ is less than $W_{N}(n)$ is removed as it is considered to be purely caused by noise. However, with this method, the inversion purely caused by instrument noise can be removed, while the effect of noise in the inversion caused by turbulence and instrument noise cannot be removed. In previous studies, lower heights were analyzed. The effect of noise can be neglected compared with the effect of turbulence. However, according to (10), when the height is greater than 30 km, with a rapid decrease of atmospheric pressure, as shown in Figure 10, the standard deviation of the noise shows a sharp increase. In this situation, it is impossible to neglect the effect of noise, which should even be taken as the main factor of inversion. The reasons mentioned above, coupled with the fact that the resolution of the instrument for stratospheric observations is not ideal enough, cause a large number of inversions with larger thickness and $L_{T}$ above 30 km.

## 5. Conclusions

After the analysis, we find that, in the tropospheric free atmosphere, our results are basically consistent with the previous research results. This shows that with Thorpe analysis combined with appropriate denoising methods to analyze the conventional balloon sounding data, the turbulent distribution and the corresponding turbulent parameters of the free atmosphere in the troposphere can be well retrieved. The difference is that because the sounding station we chose is at a low latitude (113°05′, 28°12′), the height of the tropopause is about 18 km, which is much larger than that of the tropopause (about 10 km) in the previous study [19,21]. Therefore, in our research, at a relatively large height range of 5 km to 16 km, $L_{T}$ has maintained a larger value, and ε is basically in the range of 10^−4^–10^−3^ m^2^ s^−3^. In the height range of the tropopause to about 30 km, the atmospheric environment is more stable than that of the troposphere. Therefore, the $L_{T}$ scale and the number of inversions obtained with Thorpe analysis are significantly smaller, and the inversion thickness and $L_{T}$ scale are concentrated in the range of 1–2 times the sensors’ vertical resolution (0–20 m). Because of the significant reduction of the turbulence scale in this height range, most of the turbulence is beyond the detection limit of the sensors and cannot be detected. Only a small amount of large-scale turbulence can be detected by the sensors. As the sensors can only detect large-scale turbulence, and the stratospheric buoyancy frequency is greater than that of the troposphere, the magnitude of ε obtained in this height range is roughly the same as that of the troposphere, or even slightly higher than that of the troposphere. Therefore, we believe that with Thorpe analysis, turbulence characteristics in the height range from tropopause to 30 km can be well analyzed, but it is only applicable to larger scale turbulence.

Between 30–40 km, a large part of the inversion thickness, Thorpe displacement, and $L_{T}$ are beyond normal levels. According to the analysis above, we know that, because of the rapid decrease of atmospheric pressure, the potential temperature noise will increase exponentially, which will affect the calculation of turbulent parameters. However, the inversion of this part has been tested by the denoising process, indicating that it also contains turbulence. Therefore, between 30–40 km, with Thorpe analysis, the only general distribution area of large-scale turbulence can be estimated, while the turbulence parameters cannot be calculated accurately unless we find a more accessible denoising means, which will be exactly our research work in the next stage.

## Figures and Tables

**Table 1:** The accuracy of the sensors. T—temperature; P—pressure; RH—relative humidity.

Parameter	Measuring Range	Accuracy
T	−90 °C~50 °C	ΔT ≤ ±0.3 °C (−90 °C < T < −80 °C) ΔT ≤ ±0.2 °C (−80 °C ≤ T < 40 °C) ΔT ≤ ±0.3 °C (40 °C ≤ T < 50 °C)
P	5 hPa~1060 hPa	ΔP ≤ ±1 hPa (P > 500 hPa) ΔP ≤ ±2 hPa (P ≤ 500 hPa)
RH	0 %RH~100 %RH	ΔRH ≤ ±5 %RH (T > −25 °C) ΔRH ≤ ±10 %RH (T ≤ −25 °C)

## References

[1] Theuerkauf A., Gerding M., Lübken F.J. (2011). LITOS—A new balloon-borne instrument for fine-scale turbulence soundings in the stratosphere. Atmos. Meas. Tech..

[2] Muhsin M., Sunilkumar S.V., Ratnam M.V., Parameswaran K., Murthy B.V.K., Ramkumar G., Rajeev K. (2016). Diurnal variation of atmospheric stability and turbulence during different seasons in the troposphere and lower stratosphere derived from simultaneous radiosonde observations at two tropical stations, in the indian peninsula. Atmos. Res..

[3] Gavrilov N.M., Fukao S. (2004). Turbulent diffusivity in the free atmosphere inferred from MST radar measurements: A review. Ann. Geophys..

[4] Kantha L.H. (2003). On an Improved Model for the Turbulent PBL. J. Atmos. Sci..

[5] Luce H., Fukao S., Dalaudier F., Crochet M. (2002). Strong Mixing Events Observed near the Tropopause with the MU Radar and High-Resolution Balloon Techniques. J. Atmos. Sci..

[6] Alisse J., Sidi C. (2000). Experimental probability density functions of small-scale fluctuations in the stably stratified atmosphere. J. Fluid Mech..

[7] Zhang Y., Sheng Z., Shi H., Zhou S., Shi W., Du H., Fan Z. (2017). Properties of the Long-Term Oscillations in the Middle Atmosphere Based on Observations from TIMED/SABER Instrument and FPI over Kelan. Atmosphere.

[8] Gavrilov N.M., Luce H., Crochet M., Dalaudier F., Fukao S. (2005). Turbulence parameter estimations from high-resolution balloon temperature measurements of the MUTSI-2000 campaign. Ann. Geophys..

[9] Clayson C.A., Kantha L. (2008). On Turbulence and Mixing in the Free Atmosphere Inferred from High-Resolution Soundings. J. Atmos. Ocean. Technol..

[10] Hooper D.A., Thomas L. (1998). Complementary criteria for identifying regions of intense atmospheric turbulence using lower VHF radar. J. Atmos. Sol.-Terr. Phys..

[11] Cohn S.A. (1995). Radar Measurements of Turbulent Eddy Dissipation Rate in the Troposphere: A Comparison of Techniques. J. Atmos. Ocean. Technol..

[12] Fan Z.Q., Sheng Z., Wan L., Shi H.Q., Jiang Y. (2013). Comprehensive assessment of the accuracy of the data from near space meteorological rocket sounding. Acta Phys. Sin..

[13] Li J.W., Sheng Z., Fan Z.Q., Zhou S.D., Shi W.L. (2017). Data Analysis of Upper Atmosphere Temperature Detected by Sounding Rockets in China. J. Atmos. Ocean. Technol..

[14] Zhou L., Sheng Z., Fan Z., Liao Q. (2017). Data Analysis of the TK-1G Sounding Rocket Installed with a Satellite Navigation System. Atmosphere.

[15] Thorpe S.A. (1977). Turbulence and Mixing in a Scottish Loch. Philos. Trans. R. Soc. Lond. A.

[16] Thorpe S.A. (2005). The Turbulent Ocean.

[17] Dillon T.M. (1982). Vertical overturns: A comparison of Thorpe and Ozmidov length scales. J. Geophys. Res. Oceans.

[18] Wesson J.C., Gregg M.C. (1994). Mixing at Camarinal Sill in the Strait of Gibraltar. J. Geophys. Res. Oceans.

[19] Kantha L., Hocking W. (2010). Dissipation rates of turbulence kinetic energy in the free atmosphere: MST radar and radiosondes. J. Atmos. Sol.-Terrest. Phys..

[20] Nath D., Ratnam M.V., Patra A.K., Murthy B.V.K., Rao S.V.B. (2010). Turbulence characteristics over tropical station Gadanki (13.5° N, 79.2° E) estimated using high-resolution GPS radiosonde data. J. Geophys. Res. Atmos..

[21] Wilson R., Luce H., Dalaudier F., Lefrère J. (2010). Turbulence Patch Identification in Potential Density or Temperature Profiles. J. Atmos. Ocean. Technol..

[22] Wilson R., Luce H., Hashiguchi H., Dalaudier F., Fukao S., Nakajo T., Shibagaki Y., Yabuki M., Furumoto J. Small scale turbulence and instabilities observed simultaneously by radiosondes and the MU radar. Proceedings of the International Workshop on Technical & Scientific Aspects of MST Radar 2012.

[23] Wilson R., Luce H., Hashiguchi H., Shiotani M. (2013). On the effect of moisture on the detection of tropospheric turbulence from in situ measurements. Atmos. Measur. Tech..

[24] Wilson R., Luce H., Hashiguchi H., Nishi N., Yabuki Y. (2014). Energetics of persistent turbulent layers underneath mid-level clouds estimated from concurrent radar and radiosonde data. J. Atmos. Sol.-Terrest. Phys..

[25] Balsley B.B., Kantha L., Colgan W. (2009). On the Use of Slow Ascent Meter-Scale Sampling (SAMS) Radiosondes for Observing Overturning Events in the Free Atmosphere. J. Atmos. Ocean. Technol..

[26] Alappattu D.P., Kunhikrishnan P.K. (2010). First observations of turbulence parameters in the troposphere over the Bay of Bengal and the Arabian Sea using radiosonde. J. Geophys. Res. Atmos..

[27] Thrastarson H.T., De La Torre Juarez M. Retreiving Stratopause Height from COSMIC Radio Occultations. Proceedings of the AGU Fall Meeting.

[28] Dee D.P., Coauthors (2011). The ERA-Interim reanalysis: Configuration and performance of the data assimilation system. Q. J. R. Meteorol. Soc..

[29] Businger J.A., Wyngaard J.C., Izumi Y., Bradley E.F. (1971). Flux-profile relationships in the atmospheric surface layer. J. Atmos. Sci..

[30] Mellor G.L., Yamada T. (1982). Development of a turbulence closure model for geophysical fluid problems. Rev. Geophys..

[31] Wilson R., Dalaudier F., Luce H. (2011). Can one detect small-scale turbulence from standard meteorological radiosondes?. Atmos. Meas. Tech..

[32] Gavrilov N.M., Richmond A.D., Bertin F., Lafeuille M. (1994). Investigation of seasonal and interannual variations of internal gravity wave intensity in the thermosphere over Saint Santin. J. Geophys. Res. Space Phys..

[33] Xiao C., Hu X., Gong J., Liu J. (2008). Analysis of the Characteristics of the Stratospheric Quasi-zero Wind Layer over China. Chin. J. Space Sci..

[34] Gage K.S. (1990). Radar Observations of the Free Atmosphere: Structure and Dynamics. Radar in Meteorology.

[35] Gage K.S., Green J.L., Vanzandt T.E. (1980). Use of Doppler radar for the measurement of atmospheric turbulence parameters from the intensity of clear-air echoes. Radio Sci..

